# Planning and Implementing Social Media Communication in a Public Health Crisis: An Analytical Perspective

**DOI:** 10.1002/hpja.70179

**Published:** 2026-04-05

**Authors:** Babatunde A. Balogun, A. Hogden, L. Yang, M. Agaliotis, N. Kemp

**Affiliations:** ^1^ Australian Institute of Health Service Management, University of Tasmania Sydney Australia; ^2^ School of Population Health, University of New South Wales Sydney Australia; ^3^ Tasmanian School of Business and Economics, University of Tasmania Hobart Australia; ^4^ School of Health Science, Western Sydney University Sydney Australia; ^5^ School of Psychological Sciences, College of Health and Medicine, University of Tasmania Hobart Australia

**Keywords:** COVID‐19, pandemic management plans, public communication, public health directives, public health service organisations, risks, social media

## Abstract

**Issue Addressed:**

The use of social media as a public communication tool by public health service organisations (PHSOs) gained prominence during the COVID‐19 pandemic. However, as the pandemic evolved into a prolonged public health crisis, PHSOs faced challenges in effectively engaging the public with crisis directives. Despite its growing importance, there is scant research on how PHSOs formulated and implemented their social media communication plans as part of their pandemic management.

**Methods:**

Textual analysis and quality appraisal of the first COVID‐19 pandemic management plans and the post‐COVID reports of Australia's foremost federal and jurisdictional PHSOs were undertaken. The Prevention–Preparedness–Response–Recovery framework for emergency management informed the analyses.

**Results:**

Sixteen documents were identified as relevant for inclusion in the review. Textual analysis revealed that Australia's PHSOs initially exhibited inertia in recognising social media as a strategic tool for public communication, as the documents were primarily focused on the response phase of the pandemic. The documents contained insufficient evidence of goals and metrics that could have informed how social media was used for public engagement. Jurisdictions varied in their perception of pandemic risks and in their use of social media to communicate public health directives.

**Conclusion:**

The content of the PHSOs' strategic pandemic management plans for COVID‐19 played a major role in how social media communication of public health directives was implemented.

**So What?:**

This study offers actionable insights on public health communication for pandemic management to practitioners, highlighting aspects of social media communication plans to support timely implementation.

## 1 Introduction

Effective public communication is a critical function of governance during a pandemic [1]. Governments exercise this function through their public health service organisations (PHSOs) to provide accurate, relevant, and timely information. This information can support the public to perceive risks appropriately and to adhere to health crisis directives. The World Health Organisation (WHO) recommends that countries have pandemic management plans in place with a robust strategy for public communication and engagement during public health emergencies [2]. Effective communication during a pandemic not only informs the public but also consults and includes them in the implementation of pandemic plans [3].

Global, national, and sub‐national PHSOs developed pandemic management plans specifically addressing COVID‐19, which explicitly included provisions for communication of public health directives [4]. For example, in their evaluation of COVID‐19 pandemic management plans across 106 countries, Mustafa et al. [4] found that 95% included activities related to public communication. Therefore, one could contend that most pandemic management plans were strategically sufficient for public communication. However, it appeared that the experience of PHSOs with public communication varied considerably both between [5, 6] and within [7, 8] countries. Australia was one country where disparate public communication efforts were evident among the jurisdictional PHSOs [9]. Accordingly, our first aim was to appraise the content of the COVID‐19 pandemic management plans of Australia's PHSOs relating to public communication.

PHSOs are keen to integrate social media platforms with traditional communication channels to facilitate rapid and widespread public access to information [10]. Many PHSOs steadily increased their daily social media output from their pre‐COVID levels following the COVID‐19 outbreak, which expanded public access to timely crisis directives [10]. However, key health promotion principles—such as community action and engagement—that enable people and communities to take control over the factors that influence their health [11] were not consistently achieved. Information shared through social media platforms was often unevenly accessible, culturally misaligned or insufficiently tailored to the needs of diverse communities. These limitations reinforced existing disparities and contributed to the disproportionate impacts of COVID‐19 on communities experiencing social and structural disadvantage shaped by social and digital determinants of health—such as limited language proficiency and digital literacy barriers [12]. Such inequities exposed during the pandemic thus highlight the need to understand how social media practices can support—or impede—equitable access to trustworthy, actionable health information.

Moreover, as the pandemic became a protracted public health crisis, message fatigue set in and the public progressively steered away from both engaging with COVID‐19 social media content and practising the crisis directives [13]. Further complicating this message fatigue were the inconsistencies and incongruencies in the timing, framing and transparency of PHSOs' crisis directives on social media. For example, Batova [14] found frequent discrepancies on Twitter (now X) in the messaging of the US Centers for Disease Control and Prevention (CDC) about the use of face masks, triggering public mistrust during the early stages of the pandemic. Wang et al. [8] reported further controversies generated on X in the US, as the 50 state PHSOs were at variance with one another, and with the CDC. The controversies were reflected in the context and content of their public health directives, such as handwashing and physical/social distancing. Similar disparities were reported in other countries, including Nigeria [7], Canada [15] and China [16], where PHSOs at the subnational level differed for several months in their social media communication on pandemic preventive and control measures. In Australia, inconsistencies were also evident—for example, state and territory health departments disseminated divergent advice on mask wearing, staying at home and border control during 2020–2021, reflecting broader jurisdictional differences in public health directives [17, 18].

In view of the many challenges that impacted PHSOs' social media communication of public health directives during the COVID‐19 pandemic, researchers and practitioners have called for deeper research into understanding the application of social media for effective public health communication [19]. Specifically, it is important to investigate PHSOs' implementation of their COVID‐19 pandemic plans for risk and crisis communication with the public on social media. Evidence points to PHSOs' limited success in harnessing the social media environment for effective public engagement and communication [20]. However, existing studies focus either on how PHSOs could translate their pandemic public communication plans into action [21] or on how PHSOs could address gaps in the implementation of their public communication practice [22]. There is a dearth of research that explores the connection between the public communication plans and practice. To this end, our second aim was to examine how the PHSOs' COVID‐19 pandemic management plans were formulated and translated into social media communication practices for public health directives, with a focus on identifying whether formal policies and procedures were employed.

Guided by our two aims, this study evaluated Australia's COVID‐19 management plans and post‐COVID‐19 reports at both the federal and state/territory level through textual analysis and quality appraisal to address the research question: *How did the public communication plans of Australia's PHSOs support the use of social media to communicate public health directives during the COVID‐19 pandemic?*

## 2 Methods

The COVID‐19 management plans of the Australian national government (hereafter called the Commonwealth) and the eight jurisdictions of Australia and post‐COVID reports of their foremost PHSOs were analysed. The jurisdictions are New South Wales (NSW), Victoria (VIC), Queensland (QLD), South Australia (SA), Tasmania (TAS), Western Australia (WA), the Australian Capital Territory (ACT) and the Northern Territory (NT). The plans represented what the PHSOs set out to do, while the reports described what was done. Following the approach of Briggs et al. [23], a qualitative systematic document review of the sections on public communication was conducted. This enabled us to identify strengths and gaps in the social media communication plans and policies and derive practical recommendations for improvement. Consequently, the results of the analysis of the plans and reports were compared to evaluate the execution of social media public communication of public health crisis directives. It was apparent that social media communication evolved across jurisdictions and over time during the pandemic. Supporting Information 1 presents a visual comparison of social media (Facebook) output across jurisdictions between two key periods—the first and last 30 days of their respective pandemic plans. The infographic, which demonstrates that jurisdictional PHSOs varied in their use of social media for pandemic communication, provides essential context that shaped the objectives of this study.

### 2.1 Data Sources and Collection

A desktop internet search was conducted to identify the pandemic management plans and reports of the Commonwealth and the eight jurisdictions. Relevant eligibility criteria were established to select both sets of documents for analysis. First, the plans ought to have been produced around the period that the COVID‐19 pandemic started (Supporting Information 2) and describe a national (for the Commonwealth) or jurisdictional approach to COVID‐19 pandemic management. The report must have been produced by the foremost jurisdictional PHSOs or their Chief Medical/Health Officers—as they led the pandemic response on behalf of the Commonwealth and jurisdictional governments [24]—and published after the COVID‐19 pandemic had been declared over by the jurisdiction concerned (Supporting Information 2). Neither ethics approval nor informed consent was required for this study because it did not involve human participants or animals.

### 2.2 Data Extraction and Analysis Procedures

All identified documents were screened using a standardised data extraction template (Supporting Information 3A) to determine whether they met our eligibility criteria. An initial version of the template, collaboratively developed by the authors based on the methodology described by Briggs et al. [23], was piloted on four documents and subsequently refined twice. These refinements were undertaken to ensure that the final template produced consistent outputs for our desktop review of the documents. Data collected included the following: publication information, scope of the document, evidence of public communication planning, evidence of social media use in the public communication mix, and explicit social media strategies to promote public health directives. Subsequently, analysis of all the selected eligible documents was conducted by Author 1 (BAB) in two sequential phases. To ensure reliability, the analysis was validated by Author 2 (AH), who had prior experience with qualitative data analysis software. This validation involved a comparative assessment of codes and associated child codes to evaluate inter‐coder agreement.

For the first phase, all authors jointly discussed the coding process and agreed on potential keywords to be deductively coded and analysed in alignment with the research objectives. A preliminary codebook was then drafted by Authors 1 and 2 (BAB and AH) to define these keywords, following which the full research team reviewed, revised and approved the final version. Four sets of keywords were defined, which informed our text search and word frequency evaluation:

- ‘Public communication’ to uncover how it was used to demonstrate the importance of public engagement in the documents.
- ‘Risk(s)’, ‘risk communication’, ‘crisis/crises’ and ‘crisis communication’ to evaluate the probable focus of the PHSOs' public communication initiatives.
- ‘Public health measure(s)’ and 12 specific COVID‐19 public health directives to identify the directives that the PHSOs meant for public communication and engagement.
- ‘Social media’ and all social media platforms mentioned to reveal the purpose of PHSOs' chosen channels of communication of their public health directives.

NVivo R1 software was used to text‐analyse the selected documents, specifically focusing on the social media communication plan components. The textual analysis covered text search and word frequency evaluation. We adopted the Prevention, Preparedness, Response and Recovery (PPRR) framework [25] for our textual analysis of public communication, as most countries used the four pandemic phases within the framework for their COVID‐19 pandemic management planning [26]. Thus, our analysis through the four phases of the PPRR framework assisted us in identifying the relative importance that the documents placed on each phase. As part of our reliability strategy, the remaining authors independently reviewed the codes and child codes developed by Authors 1 and 2 (BAB and AH), after which the full research team convened to discuss and resolve any discrepancies through consensus.

For the second phase, an evaluation tool to support the quality assessment of the social media communication plan component of the selected COVID‐19 management plans and the post‐COVID reports was collaboratively developed by the authors (Supporting Information 3B). The tool, which was based on the Barcelona Principles 3.0 framework [27], provided a comprehensive set of metrics and guidelines for effectively evaluating social media public communication efforts of organisations [28]. Our evaluation tool contained a scale of nine domains that incorporated the evaluation criteria established in public document review literature [23, 28]. The domains were: background and ‘case for social media’, public opportunity, goals, stakeholder orientation, time allocation, measurement and evaluation, obligations, resource considerations and integrity and transparency. They were used to score each document on a 3‐point nominal scale (0–2, for a maximum score of 18). The evaluation tool was applied by Author 1 (BAB) to all the documents. To ensure validity and reliability, the remaining authors independently reviewed the assessment process, and all discrepancies were addressed during a team meeting. The quality assessment process highlighted the strengths and weaknesses of the selected documents.

## 3 Results

### 3.1 Overview of Selected Documents

**FIGURE 1:**
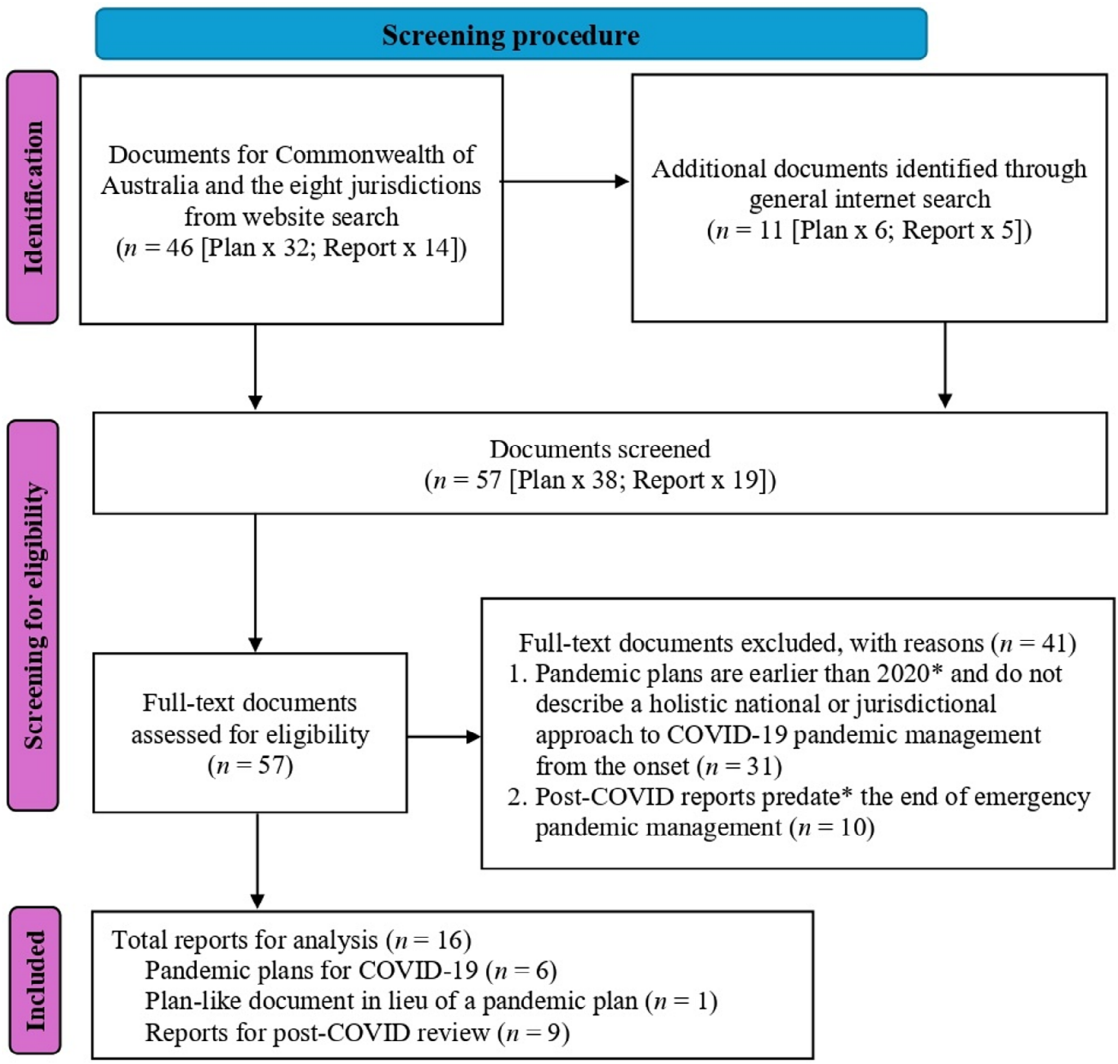
Search flow diagram of eligible COVID‐19 documents (PRISMA template). *Eligibility criterion waived for one pandemic management plan and two post‐COVID reports.

Our search identified 57 documents, comprising 38 pandemic management plans and 19 pandemic management reports (see Figure 1). At screening, based on our eligibility criteria and the results of the standardised data extraction process, 41 documents were removed. Thus, 16 documents, comprising six pandemic management plans, one substitute document for a plan and nine post‐COVID reports, were included in our final analysis. The ACT, NT and TAS had no relevant publicly available pandemic management plans for COVID‐19, but we found a substitute document of TAS sufficiently relevant to use in lieu of a pandemic management plan, as it contained detailed plan‐like operational updates. NSW's pandemic management plan did not fully meet the eligibility criteria, as it was not COVID‐19 specific; but it was included in our analysis, as it was the document that underpinned the NSW COVID‐19 response [29]. In terms of the nine post‐COVID reports, there was no publicly available relevant document of the Commonwealth and TAS that fully met the eligibility criteria as of the time of our data collection; instead, we found substitute reports for the analysis.

### 3.2 Content of the Pandemic Management Plans and Post‐COVID Reports

Our first aim was to appraise the content of the COVID‐19 pandemic management plans of Australia's PHSOs relating to public communication and summarise the inclusion of a public communication plan by document (Table 1). In relation to the PPRR framework [25], most pandemic management plans (85.7%) described public communication aim/purpose and strategies for the response phase, either alone or together with one other phase—most commonly the preparedness phase. Only WA's plan contained strategies that covered all four phases. Similarly, most post‐COVID reports (77.8%) focused on the response phase only. The reports of the Commonwealth and QLD did not mention a public communication strategy or purpose.

**TABLE 1:** Inclusion of a public communication plan in analysed documents.

Authority	Document title (publication date)	Description of strategy	Purpose or aim of strategy (direct quotes)	Pandemic phases included within scope
COVID‐19 pandemic management plans				
Commonwealth	Australian Health Sector Emergency Response Plan for Novel Coronavirus (COVID‐19) (18 February 2020)	Explicit	*Public communication will be used to provide an opportunity both to address any public concern caused by the novel coronavirus outbreak and to engage the public in strategies to manage the impact of the disease*	Response phase of pandemic
NSW	NSW Human Influenza Pandemic Plan: A Sub Plan of the NSW State Emergency Management Plan (7 June 2018)	Implicit	*Providing the public with relevant and current information is a key priority for the NSW Government during a pandemic. … aid in limiting the spread of disease and the potential for elevated anxiety levels within the community*	Preparedness and response phases of pandemic
QLD	Queensland Whole‐of‐Government Pandemic Plan (March 2020)	Explicit	*The purpose of the Queensland Whole‐of‐Government Pandemic Communication Plan is to provide a strategic communication framework for the Whole‐of‐Government (WoG) response to an influenza pandemic, in support of Queensland Health (QH) as the lead agency*	Unstated
SA	SA Health Viral Respiratory Disease Pandemic Response Plan (including influenza, COVID‐19, SARS & MERS) (March 2020)	Explicit	*Communication with the public, through the media and other sources, will shape the public perception of risk and public engagement in measures to address the pandemic*	Preparedness and response phases of pandemic
TAS	Department of Health Submission to the Independent Review of the Response to the North‐West Tasmania COVID‐19 Outbreak: Communication (1 September 2020)	Explicit	*… open and transparent communication with the wider community has provided clear advice on restrictions and measures to prevent further transmission of COVID‐19*	Preparedness and response phases of pandemic
VIC	COVID‐19 Pandemic plan for the Victorian Health Sector (10 March 2020)	Explicit	*DHHS has developed a communications plan to help encourage Victorians to take proactive measures to minimise disease transmission*	Response phase of pandemic
WA	Western Australian Government Pandemic Plan (11 March 2020)	Explicit	*Clear, accurate and timely information is essential to support communities. It's important the public understand the current situation, risks and what they need to do to protect themselves*	All phases of pandemic (prevention, preparedness, response and recovery)
COVID‐19 pandemic reports				
Commonwealth	Chief Medical Officer's Report (report; September 2022)	No	Not provided	Unstated
ACT	ACT Chief Health Officer's Report on the COVID‐19 Public Health Emergency (14 November 2023)	Explicit	*It was recognised early that Canberrans required a single source of reliable information about the local situation and the protective behaviours that should be adopted*	Response phase^a^ of pandemic
NSW	Public Health—NSW COVID‐19 Response (April 2023)	Explicit	*Empowering the public with the right information was critical for the response. … and help the community understand the behaviours needed from individuals, communities and organisations to prevent the spread of disease during pandemics*	Response phase^a^ of pandemic
NT	Chief Health Officer's Report: The COVID‐19 Public Health Emergency (16 September 2022)	Explicit	*The Territory government's public information strategy provided frequent, factual, consistent and clear messaging delivered by trusted health and community leaders, on platforms and in languages to suit the diverse needs of Territorians*	Response phase of pandemic
QLD	The health of Queenslanders: Report of the Chief Health Officer Queensland (March 2023)	No	Not provided	Unstated
SA	The Chief Public Health Officer's Report 2020–2022 (2023)	Explicit	*Throughout the COVID‐19 response, it was critical for the community to have up‐to‐date, clear and consistent information around COVID‐19 advice and requirements*	Response phase^a^ of pandemic
TAS	Report of the Auditor‐General (23 March 2021)	Implicit	*Overall, communication and information was generally effective in supporting decision‐making and external communication with the public was adapted quickly to broadly inform and protect the Tasmanian community*	Response phase of pandemic
VIC	Review of COVID‐19 Communications in Victoria (September 2022)	Explicit	*Due to the unprecedented and dynamic nature of the COVID‐19 pandemic, Victorians needed clear and consistent information on COVID‐19 and pandemic orders to understand risks and current requirements to comply with*	Response phase of pandemic
WA	Review of Western Australia's COVID‐19 Management and Response (July 2023)	Explicit	*Public communications were a critical component of the WA Government's response, with the Premier's daily updates and the centralised communications in the Department of the Premier and Cabinet praised for creating a single source of trusted information*	Preparedness, response and recovery phases of pandemic

^a^ The document did not describe phase using the PPRR framework [25].

The results of our NVivo text search and word frequency analysis are presented in Table 2. The term ‘public communication(s)’ was used to signal the importance of public engagement. Yet it appeared only 12 times in total, and only in three of the seven pandemic management plans (Commonwealth, NSW and WA), in each case referring specifically to the response phase of the pandemic. Six post‐COVID reports mentioned the term 19 times, and consistent with the pandemic management plans, the term was primarily associated with the response phase. However, whereas all mentions in both plans and reports emphasised the critical role that public communication was expected to play during the pandemic management, only once was the term defined and clarified. Thus, the term was left open to subjective interpretation.

Public communication is … the real‐time provision of information and advice to support individuals and groups to make informed decisions and minimise their risk. In addition to specific risk communication approaches, standard responsive public communications are also used to provide advice … (ACT report, p. 15)

**TABLE 2:** NVivo text search and word frequency analysis.

Texts in documents	Word counts	Number of plans	Word coverage (%)
Plans			
Prevention	72	7	0.44
Preparedness	140	7	0.85
Response	666	7	2.86
Recovery	151	7	0.86
Public communication(s)	12	3	0.12
Risk(s)	256	7	0.53
Risk communication(s)	2	2	0.02
Crisis/crises	59	4	0.41
Crisis communication(s)	10	1	0.22
Public health measure(s)	16	2	0.13
Social media	27	6	0.20
Reports			
Prevention	132	8	0.35
Preparedness	89	8	0.24
Response	1770	9	2.78
Recovery	94	8	0.16
Public communication(s)	19	6	0.08
Risk(s)	825	8	0.58
Risk communication(s)	29	4	0.11
Crisis/crises	52	6	0.08
Crisis communication(s)	9	1	0.04
Public health measure(s)	42	6	0.12
Social media	97	8	0.22

Evaluating the focus of the PHSOs' public communication initiatives, our analysis revealed a consistent framing of the pandemic as a risk rather than a crisis. All seven pandemic management plans mentioned ‘risk(s)’ (mean weighted coverage: 0.08%), while only four mentioned ‘crisis/crises’ (0.06%). Notably, two plans (NSW and QLD) cited ‘crisis/crises’ three times more often than ‘risk(s)’, indicating a jurisdiction‐specific inclination towards crisis‐oriented framing of the pandemic. Meanwhile, ‘risk communication(s)’ was mentioned once in each of two plans (Commonwealth and SA) only, and ‘crisis communication(s)’ appeared in only one plan (QLD); in all instances, both terms were linked to the intended public communication activities within their jurisdictions. Altogether, although all the plans articulated the need for both pandemic risk and crisis mitigation, public communication was not positioned as a central priority.

In the post‐COVID reports, eight of nine mentioned ‘risk(s)’ (mean weighted coverage: 0.06%), but only six referenced ‘crisis/crises’ (0.01%). Meanwhile, four post‐COVID reports (ACT, NSW, VIC and WA) collectively mentioned ‘risk communication(s)’ 29 times, whereas only VIC report mentioned ‘crisis communication(s)’. The mentions of ‘risk communication(s)’ and ‘crisis communication(s)’ were typically associated with actual public communication activities, such as *creat*[ing] *a sense of urgency for COVID‐19 vaccination* (ACT report, p. 104) and *engag*[ing] *with a wide range of marginalised populations in relation to health information* (NSW report, p. 149). Notably, the VIC report was the only document to employ relevant theoretical communication frameworks to buttress the importance of ‘risk communication(s)’ and ‘crisis communication(s)’ in a pandemic context, drawing on Sandman's risk communication model, CDC's Crisis and Emergency Risk Communication model and WHO's Risk Communication and Community Engagement framework.

Overall, ‘risk(s)’ appeared 10 times more frequently than ‘crisis/crises’ across all the documents, underscoring a dominant risk‐oriented narrative in the PHSOs' pandemic‐related activities throughout the course of the pandemic. Despite this emphasis, specific descriptions of what risk communication covered were limited to the following: ‘to provide appropriate advice to the community’ and ‘to establish confidence and trust’ (ACT report, p. 25), ‘to address growing community fatigue with remaining COVID‐19 restrictions’ (ACT report, p. 104) and ‘to proactively address counter‐responses including myths’ (VIC report, p. 23). Risk and crisis communication were generally neither consistently planned nor systematically implemented in the public communication efforts of Australia's PHSOs across all the COVID‐19 pandemic phases. For example, of the combined 50 mentions of ‘risk communication(s)’ and ‘crisis communication(s)’, only seven documents included both terms, with 11 mentions directly associated with ‘response’ but none linked to the other three pandemic framework terms. Although all mentions underscored the vital contribution that both forms of communication (was expected to) make to pandemic management, the terms were clearly defined in one document only—leaving them open to potentially inconsistent interpretation.

Risk communication is communication intended to provide audience members with the information they need to make informed, independent judgements about risks to health, safety, and the environment. (VIC report, p. 23)

Crisis communication … is more typically associated with public relations after a crisis or disaster. For this reason, crisis communication has traditionally formed part of emergency management models and disaster response. (VIC report, p. 23)

Overall, the conversations about public communication, risk communication and crisis communication messages were directed at specific key target audiences in four plans and five reports. The audiences included businesses, public transport systems, schools, First Nations people, culturally and linguistically diverse population, people living with disability and aged care communities, among others.

### 3.3 Execution of Social Media Communication of Public Health Directives

Our second objective was to identify and evaluate the possible relationships between PHSOs' COVID‐19 pandemic management plans and execution of social media public communication of public health directives. Our textual analysis (Table 2) showed that only two pandemic management plans (Commonwealth and SA) mentioned the term ‘public health measure(s)’. However, both plans used the term quite differently. The Commonwealth plan explicitly underlined the importance of and sought the public's active engagement and involvement for the measures to be effective. The SA plan viewed public health measures as tools for human movement control across its jurisdictional border and for the health sector practitioners to contain the pandemic. Meanwhile, six post‐COVID reports mentioned ‘public health measure(s)’. The general narrative in those reports pointed to governments as being responsible for putting public health measures in place, with the people having minimal involvement beyond compliance. Taken together, only half of the documents mentioned the term ‘public health measure(s)’. The two plans (Commonwealth and SA) accounted for 27.6% of all mentions, while 28.6% of mentions in the six reports were from the NSW report.

**FIGURE 2:**
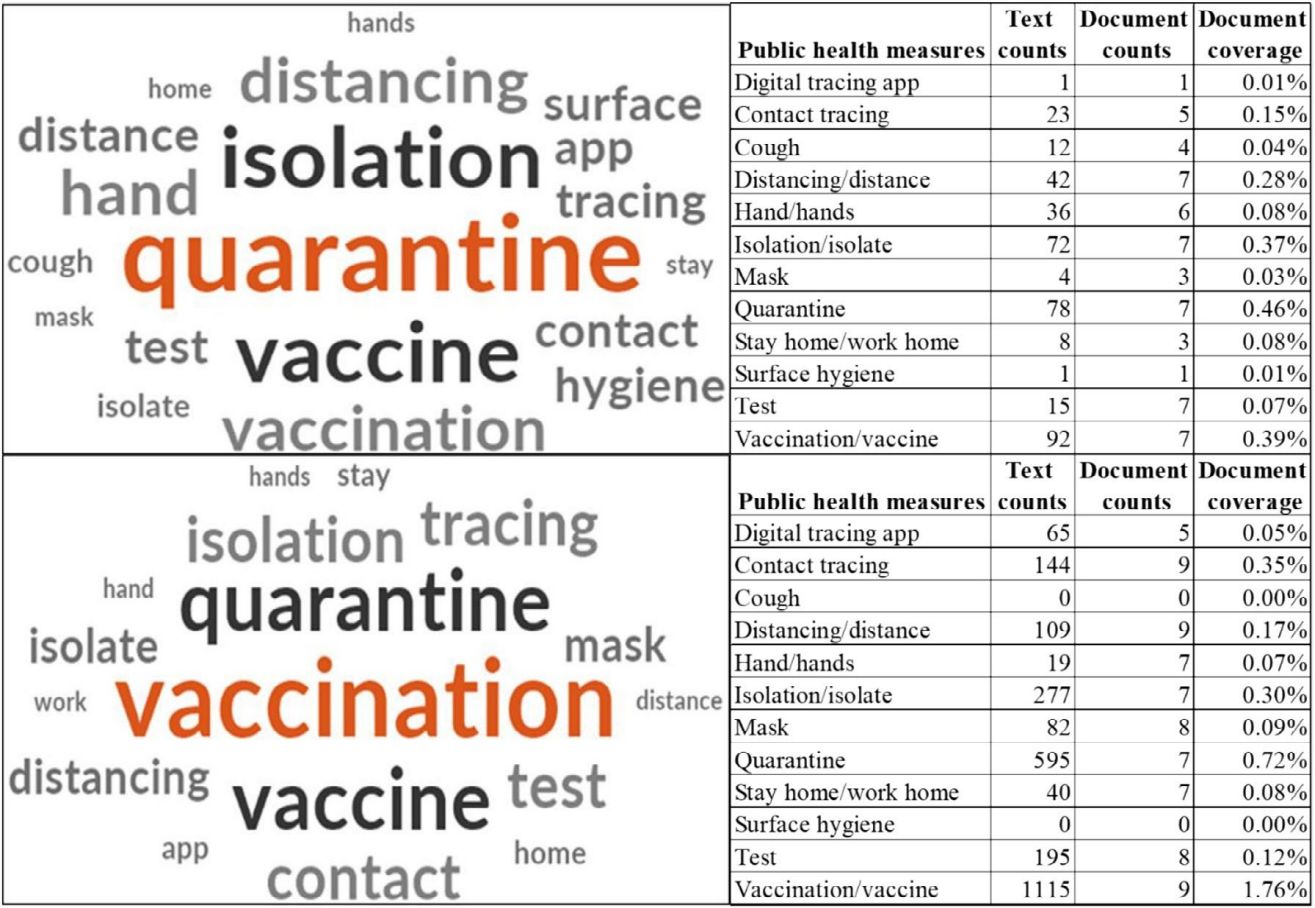
Public health directives discussed in the pandemic management plans (top) and post‐COVID reports (bottom).

Notwithstanding the use of the term ‘public health measure(s)’, all documents highlighted a range of public health directives (to be) issued and promoted during the pandemic. Overall, 12 directives: 11 non‐pharmaceutical and one pharmaceutical, were discussed in both sets of documents. The non‐pharmaceutical directives were ‘digital tracing app’, ‘contact tracing’, ‘cough’, ‘distancing/distance’, ‘hand/hands’, ‘isolation/isolate’, ‘mask’, ‘quarantine’, ‘test’, ‘stay home/work from home’ and ‘surface hygiene’. ‘Vaccination/vaccine’ was the only pharmaceutical directive. The 12 public health directives and the grouped findings from the pandemic management plans and post‐COVID reports, respectively, are represented in an NVivo Word Cloud (Figure 2). In the seven plans collectively, the main public health directives highlighted were ‘vaccination/vaccine’ (24.0%), ‘quarantine’ (20.3%) and ‘isolation/isolate’ (18.8%), while ‘digital tracing app’ and ‘surface hygiene’ appeared only in the pandemic management plans of TAS and VIC, respectively. The nine post‐COVID reports collectively concentrated on ‘vaccination/vaccine’ (42.2%), ‘contact tracing’ (5.5%) and ‘physical/social distancing/distance’ (4.1%) and there was no mention of ‘cough’ and ‘surface hygiene’ in any report. Directives such as ‘quarantine’ (22.5%) were frequently mentioned directives but did not feature in all the reports. Overall, of the 12 public health directives identified, a quarter of all directive references in the pandemic management plans related to vaccination—the only pharmaceutical directive—whereas in the post‐COVID reports, pharmaceutical and non‐pharmaceutical directives were referenced in equal volume. Worthy of note were the mentions of ‘mask’ in the pandemic management plans, which addressed health workers only, whereas the mentions applied to both health workers and the public in the post‐COVID reports.

Our findings also revealed that the jurisdictions varied in both the specific public health directives they prioritised and the volume of those directives they planned and executed in their public communications. For example, among the pandemic management plans, TAS and VIC each covered 10 public health directives, with TAS placing greatest emphasis on ‘quarantine’, followed by ‘contact tracing’ and VIC prioritising ‘isolation/isolate’ as its leading directive, ahead of ‘quarantine’. In contrast, NSW and QLD each addressed only six directives: NSW focused mainly on ‘quarantine’, followed by ‘distancing/distance’, while QLD emphasised both ‘vaccination/vaccine’ and ‘isolation/isolate’ as its primary directives. The post‐COVID reports showed that the ACT, VIC and WA each addressed 10 public health directives, while the Commonwealth focused on only four. Although all jurisdictions except TAS paid substantial attention to ‘vaccination/vaccine’, only the Commonwealth, QLD, NSW and VIC identified it as their predominant directive. The ACT, WA and SA adopted a two‐pronged approach, pairing ‘vaccination/vaccine’ with ‘quarantine’. A similar multi‐faceted approach employed by the NT combined ‘vaccination/vaccine’ with ‘contact tracing’ and ‘digital tracing app’. TAS, however, principally focused on ‘contact tracing’. The details can be found in Supporting Information 4.

To reveal if and why PHSOs incorporated social media into their public communication mix for their public health directives, we searched for the term ‘social media’ in the documents. Table 3 presents the results of our search. No pandemic management plan detailed a clear and specific strategy for social media use. About half of the plans (Commonwealth, QLD and TAS) mentioned the readiness of PHSOs to utilise Facebook, X, YouTube, Instagram and LinkedIn for social media communication; the remaining plans made no such provisions, although SA did designate local governments as responsible for disseminating information via social media. Only the plans of the Commonwealth, TAS and WA acknowledged that social media communication could pose certain risks and challenges during the pandemic, for example, ‘social media will spread messages and accentuate fear amongst all age groups’ (WA plan, p. 5).

**TABLE 3:** References to social media in PHSOs' public communication plans.

Documents	Total mentions	Intention to use	Examples cited (page)
Plans			
Commonwealth	14	Yes	*… use the Department of Health's existing social media accounts … to provide up to date notifications on … novel coronavirus outbreak information; …* (p. 34)
NSW	0	No	Not available
QLD	7	Yes	*A proactive approach to providing general updates about the issue/crisis is taken with all external communication, including social media*. (p. 23)
SA	1	No	*Local governments' role to be guided by SA Health*. (p. 16)
TAS	3	Yes	*Certain existing strategies proved invaluable to the COVID‐19 response, such as the use of social media and the Public Health Hotline*. (p. 28)
VIC	1	No	Not applicable^a^
WA	1	No	Not applicable^a^
Reports			
Commonwealth	0	No	Not available
ACT	13	Yes	*Throughout this period, communications teams focused on delivering clear public information … using … ACT Health social media channels …* (p. 98)
NSW	18	Yes	*They were streamed live by NSW Health through social media channels and were made available to view on the … social media channels afterwards*. (p. 115)
NT	3	Yes	*The Territory Government held almost daily press conferences, issued daily COVID‐19 media releases and social media posts during the emergency period*. (p. 32)
QLD	1	No	Not applicable^a^
SA	4	Yes	*Research … showed the SA Health … social media channels continued to be consistently used as key sources of information on COVID‐19, …* (p. 95)
TAS	2	Yes	*… the DoH website (including its social media accounts), … were used as early communication tools*. (p. 31)
VIC	47	Yes	*Documents that summarised significant changes to pandemic orders and public health advice … shared on social media…* (p. 31)
WA	9	Yes	*Over 80 public communications campaigns were developed by the COVID‐19 Communications team and disseminated through the … social media*. (p. 58)

^a^ Not applicable = Mention of social media was not related to any use for public communication.

Among the post‐COVID reports, all except two (Commonwealth and QLD) narrated how social media was employed for communication during the pandemic. These seven reports summarised a range of approaches and tactics that the PHSOs adopted to disseminate pandemic‐related information through their social media accounts. Such strategies discussed included the activities of a dedicated communications team (ACT, NSW, WA), content of social media messages (ACT, NSW, NT, SA, TAS, VIC, WA), specific social media platforms used (ACT, NSW, VIC, WA), targeting of specific demographic and socio‐cultural groups (ACT, NSW, NT, VIC, WA), use of in‐language (ACT, NSW, NT, VIC, WA), aiming for specific periods or key dates (ACT, NSW, NT, VIC), countering of false information (NSW, NT, VIC) and aiming to achieve specific outcomes (ACT, SA, WA). Overall, there was a discernible trend of improvement in the PHSOs' use of social media for public communication.

### 3.4 Quality Appraisal of the Social Media Communication Plan Component of the Analysed Documents

The content quality of the social media communication plans is summarised in Table 4. Using the Barcelona Principles 3.0 framework [26] as an evaluation tool, information to support the implementation of social media public communication during the pandemic was not evident in the COVID‐19 management plans belonging to five jurisdictions, namely NSW, QLD, SA, VIC and WA. The other two jurisdictions (Commonwealth and TAS), however, provided partial information about strategies to support the implementation of social media public communication, covering seven and four domains, respectively. None of the plans justified their case for using social media or provided opportunity to the public to actively participate in co‐creating and disseminating information. Only one plan (TAS) demonstrated full consideration for the measurement and evaluation of the PHSO's social media activities. Among the reports, two (Commonwealth and QLD) did not provide information on any of the nine domains. The VIC report, however, rated strongly in three domains, namely ‘business case’, ‘public opportunity’ and ‘stakeholder orientation’.

**TABLE 4:** Quality appraisal of the social media communication plans of Australia's jurisdictional PHSOs.

Domains^a^	Pandemic plans							Pandemic reports								
	COM	NSW	QLD	SA	TAS	VIC	WA	COM	ACT	NSW	NT	QLD	SA	TAS	VIC	WA
A: Background and ‘case for social media’																
The introduction section, justifying social media communication, references																
evidence from contemporary literature and	0	0	0	0	0	0	0	0	0	1	0	0	0	0	2	0
(citation of) compelling scientific grounds and																
verifiable sources of data or authorities																
B: Public opportunity																
There is evidence that the plan																
is informed by meaningful consultation (e.g., surveys, polls) with a broad range of stakeholders and	0	0	0	0	0	0	0	0	0	2	0	0	0	0	2	1
integrates evidence from consultation outcomes.																
C: Goals																
The goals/objectives of the plan are																
explicitly stated and	1	0	0	0	0	0	0	0	1	1	1	0	1	0	1	1
tangible enough to be evaluated and																
oriented towards engaging the public and to persuade adoption of preventive measures																
D: Stakeholder orientation																
The plan identifies/recognises																
target audience, including specific groups (e.g., First Nations, CALD, PLWD) and	1	0	0	0	0	0	0	0	1	1	1	0	1	0	2	1
communication channels to reach each target audience and																
communication needs of each target audience and																
diversity in health literacy levels of the target audience and																
specific metrics for each target audience and																
potential conflicting sources of information that the target audience may access and																
target audience as not passive hearers but active stakeholders, and																
opportunities to consult various target audience for the co‐design of tailored messaging																
E: Time allocation																
The plan outlines																
a probable calendar that schedules messaging and other campaigns and	1	0	0	0	1	0	0	0	1	1	0	0	0	0	1	1
time for different categories of messaging (e.g., situational information, important health messages, updates of case numbers and deaths) and																
time for listening and interacting with the public (e.g., enquiries, polls, competitions) and																
time for reporting the performance of social media activities (e.g., daily, weekly)																
F: Measurement and evaluation																
The plan considers																
the outputs based on quantitative metrics (e.g., reach, likes, shares, views) and	1	0	0	0	2	0	0	0	1	1	0	0	1	1	1	1
the outcomes based on qualitative metrics (e.g., comments, sentiments) and																
the potential impact based on public awareness, attitude or opinion data and																
the frequency of measuring outputs, outcomes and impact (e.g., daily, weekly, monthly) and																
•he reporting of measured outputs, outcomes and impact (e.g., daily, weekly, monthly)																
G: Obligations																
The plan explicitly specifies and assigns																
roles and responsibilities of implementers of activities (e.g., messaging, measuring and evaluation, listening and interacting)	1	0	0	0	1	0	0	0	0	1	1	0	0	0	0	1
H: Resource considerations																
The policy considers																
the provision for funding of social media messaging and	1	0	0	0	1	0	0	0	1	0	0	0	0	0	1	1
sourcing of spokespersons for social media messaging and																
sourcing of social media influencers for social media messaging																
J: Integrity and transparency																
The plan reveals																
the estimated resources supporting social media messaging (e.g., campaigns produced by hired external parties) and	1	0	0	0	1	0	0	0	0	1	1	0	0	0	1	1
mitigation efforts to manage the potential risks associated with communicating about an evolving public health challenge and																
human resource competences and organisational capacity to implement the plan and																
procedures and guidelines that inform how the public should use the organisation's social media platforms.																

Abbreviations: CALD, culturally and linguistically diverse population; COM, commonwealth; PLWD, people living with disabilities.

^a^ Assessment criteria (numbers reflect the degree to which a criterion is applied or addressed in the documents) = Fulfilled or strong (2); Room for improvement (1); Not fulfilled or weak (0).

## 4 Discussion

This research reports on the analysis of the content of Australia PHSOs' public communication plans and how they supported the use of social media for communicating public health directives during the COVID‐19 pandemic. The PHSOs were initially hesitant to consider social media as a strategic public communication tool, a pattern traceable to communication components of the pandemic management plans that were predominantly oriented towards the response phase. To our knowledge, this study is the first to examine COVID‐19 social media communication by analysing the content and execution of national and subnational pandemic management plans. This section explores the implications of our findings, highlights potential gaps in policy and practice, and proposes actionable recommendations for stakeholders to consider.

This study showed that most of the pandemic management plans explicitly included public communication as part of their holistic approach to managing the COVID‐19 pandemic. This finding is consistent with Mustafa et al.'s [4] survey results of pandemic management plans from 106 countries. Australia's pandemic management plans scheduled certain pandemic‐related interventions to start during the first pandemic phase or span the entirety of the four pandemic phases within the PPRR framework. However, the role of public communication was largely limited to the response phase. Additionally, most jurisdictions introduced/activated their pandemic management plans after recording COVID‐19 cases, leaving little room to plan for and disseminate risk awareness information to the public (Supporting Information 5). Our findings are consistent with those of Basseal et al. [30], pointing to the inadequacy of information dissemination and public engagement at the onset of the COVID‐19 pandemic in Australia due to the weakness in the public communication strategies outlined in pandemic management plans. No campaign alerted the public to protective measures until weeks after the outbreak [31]. A structured communication plan with unambiguous directives from the PHSOs to guide healthcare service providers and workers was also lacking. At times, there were conflicting guidelines across PHSOs, so that service centres with facilities in different jurisdictions struggled to cope with the string of conflicts [32], such as guidelines on wearing a face mask in public. Having a strategic public communication plan in place prior to a pandemic is the bedrock for meaningful public engagement [33]. Otherwise, PHSOs' capacity for effective communication could be hampered. While the existence of these plans does not guarantee success [34], it enhances the capacity of PHSOs to articulate their pandemic narratives and crisis directives with greater clarity and efficiency—contributing to more coordinated and productive outcomes across all the pandemic phases [35, 36].

Noteworthy from our analysis of the pandemic management plans was the governments' high perception of risks regarding the pandemic. Such a high level of risk perception was expected to translate into increased robustness of public communication plans and the comprehensiveness of plan implementation, including the clarity about the specific key target audience and the strategies employed to monitor the public's acceptance and responsiveness to risk communication [3, 37]. However, our analysis revealed a counterintuitive trend among Australian jurisdictions: while pandemic risk/crisis recognition was high, the emphasis on risk/crisis communication was notably low in the documents assessed (Table 2). Lim et al. [38] suggested that achieving widespread adotion of public health directives ‐ and thereby keeping mortality and morbidity rates under control ‐ required alignment between government and public perceptions of COVID‐19‐related risks. However, since how that risk is perceived varies between people from different cultural backgrounds [38], it is critical to distinguish target recipients of risk information and tailor the conversation to suit their sociocultural context. The failure of governments to adapt public communication planning and implementation to specific target audiences can lead to disparity in accessibility and compliance [5, 39]. Studies show that certain population groups such as Indigenous peoples [40] and first‐generation migrants [41] were placed at a disadvantage in navigating the COVID‐19 pandemic in Australia. System‐level shortcomings, such as those arising from the application of blanket measures and the dissemination of generic, non‐co‐designed messages, failed to meet their needs, contributing to poorer health outcomes [40, 41]. Information environment—which aggregates the public, organisations and systems involved in the collection, processing, dissemination or execution of information—is a critical social determinant of health, and PHSOs must ensure that their pandemic communication promotes health equity [42]. Pandemic management plans should clearly articulate how PHSOs intend to communicate and engage with people in a culturally appropriate manner [43]. As a practical recommendation, PHSOs should act with clearer intention, not only in their efforts to mitigate pandemic risks within their jurisdictional capacities, but also in how they actively collaborate with the public through effective communication that promotes mutual risk awareness.

Our results revealed evidence of a wide variation in the emphasis on public health directives by the PHSOs, although the pandemic management plans were drafted with the main inputs coming from the same collectively developed and unanimously endorsed source—the *2019 Australian Health Management Plan for Pandemic Influenza* (AHMPPI) [44]. This diversity has been attributed to the political structure of the country, where each jurisdiction is a federated unit with oversight of its own public health system [32]. On the one hand, this approach is argued to be essential for effective public communication of the directives in ways that are commensurate with local risks and prevailing circumstances [24]. On the other hand, the experience of poor national coordination of public communication underscores the importance of a strong national body with the expertise and resources to more proactively manage pandemics that threaten the whole country [30]. Without such a body, disjointed policy implementation at subnational levels would undermine public communication [7, 45]. Evidence from Latin America suggests that subnational policies complement but do not adequately replace a nationally coordinated pandemic management process [46]. As argued by Ihekweazu et al. [37], higher‐level coordination fosters keen participation and engagement down to the local community level. Australia has now created a national CDC [47], aiming to improve the country's preparedness for, and management of, potential future pandemics [48].

Nevertheless, the setup of such a national body is not a guarantee of better pandemic management, evidenced by the COVID‐19 experiences of some Western countries that have analogous institutions [49]. Although an independent Inquiry into the Australian Government COVID‐19 pandemic response identified significant coordination deficits in Australia's pandemic communication and recommended the establishment of a national CDC [50], there is a notable absence of publicly documented, jurisdiction‐specific evaluations assessing how such an institution might have mitigated disparities in public health directives. The federated nature of Australia's health governance—where jurisdictional PHSOs operate with considerable autonomy—resulted in heterogeneous directives, particularly in their emphasis, scope and frequency [51]. Despite these divergences, no jurisdictional PHSO has released a formal retrospective analysis examining its own coordination challenges or the potential utility of a national public health authority. This lack of critical reflection at the subnational level constitutes a missed opportunity to inform the CDC's institutional design with insights grounded in operational realities. Moreover, it raises questions about the extent to which lessons from the pandemic are being systematically internalised across Australia's multi‐level public health architecture.

Our findings also showed that only half of the pandemic management plans identified social media as an important part of the public communication mix for the PHSOs to disseminate information and engage the community. Further, none of the plans detailed a clear and specific strategy for social media use despite all Australia's PHSOs having started disseminating COVID‐19‐related information through their social media accounts before the publication of their pandemic management plans (Supporting Information 5). It is unclear, therefore, why the opportunity to outline comprehensive social media strategies in their pandemic management plans was not utilised. Extant literature has demonstrated that social media communication is invaluable during pandemics [10]. Social media platforms provided avenues for Australia's PHSOs to disseminate relevant public health directives during the COVID‐19 pandemics [9]. However, harnessing the potentials of social media for public health communication requires *a strategic communication plan that incorporates best practices for expanding reach and fostering interactivity and engagement* [33] (p. 6). Pandemic management plans should comprehensively provide for social media public communication protocols in a standardised format, with a focus on public participation in both planning and implementation. For example, PHSOs could plan to co‐design pandemic‐related messages with the public on social media. This initiative would provide the people with a sense of ownership and commitment to the public health directives, as seen in Spain, Germany and Estonia [52]. PHSOs could also identify ambassadors and influencers among their social media followers and engage them more closely as spokespeople [19].

Our evaluation of the post‐COVID reports indicated that the PHSOs demonstrated agility by gradually shifting towards strategic use of social media for public communication. This was evident even among PHSOs that had no defined social media strategy in their pandemic plans (Table 3). As the pandemic evolved, they developed clearer definitions for their activities, thereby enhancing their capacity to leverage social media in promoting public health directives. For example, VIC exclusively dedicated their Facebook page to pandemic‐related communication within the first month of activating their pandemic plan. SA continued to maintain a high frequency of Facebook usage in the final month before the winding down of its pandemic management plan, comparable to levels observed during the initial activation period (Supporting Information 1). These actions reinforce Greer's [53] arguments that PHSOs should be responsive during public health emergencies, rather than being hindered by bureaucratic constraints and insufficiently adaptive pandemic plans. Nevertheless, the post‐COVID reports mirrored the pandemic management plans in their predominant focus on the pandemic's response phase. This alignment suggests that the content of the plans significantly influenced their implementation. As a practical recommendation, PHSOs should maintain agility and adaptability in responding to unfolding public health emergencies, unencumbered by inflexible pandemic management plans.

Additionally, our findings revealed an apparent disparity among the PHSOs in their risk observation levels and the public health directives that they promoted. Broad divergence of pandemic management efforts within a country often creates the perception of incoherence to the public [8, 17]. Eastwood et al. [54] suggested that Australians had a high willingness to comply with physically and socially disrupting public health directives during a pandemic, and this willingness increased with a higher perception of risk. Therefore, there may have been a missed opportunity to ensure that the people did not become disenchanted or unsatisfied, especially during the prevention and preparedness phases of the COVID‐19 pandemic.

Our quality appraisals demonstrated a general information deficiency about the metrics and guidelines used by PHSOs to assess the effectiveness of their social media public communication efforts. The public communication plans of Australia's PHSOs for the COVID‐19 pandemic contained insufficient evidence to indicate that social media was meant to be a strategic tool for communicating public health directives. This finding was unsurprising given that most of the pandemic management plans did not articulate an intention of the PHSOs to use social media for public communication. We argue that this lack of declaration of intention contributed to the information deficiency. Additionally, the lack of declaration of intention also accounted for the paucity of clear key performance indicators related to the reach of the PHSOs' messaging and the level of public engagement with their messaging. Performance indicators are essential for applying metrics to monitor their effectiveness and measure the impacts and outcomes of social media public communication efforts [55]. Public sector communication is goal‐oriented, aiming to establish and maintain public good and engender mutual trust between the state and the public [56]. Therefore, it should be designed not merely to ask the public to ‘lend their ears’ but also to ‘have their say’. This stance becomes even more pertinent during a pandemic when the public seek trusted sources to communicate with. By addressing the deficiencies identified, Australia's PHSOs could find social media platforms to be effective strategic tools to shape public opinion about public health directives [57, 58]. Early commencement of social media messaging by PHSOs is helpful to support the public in making informed decisions about self‐ and community protection during pandemics [10]. However, prompt social media messaging during a public health crisis requires prior comprehensive planning coupled with appropriate resources and personnel. For example, Steffens et al. [59] observed that health organisations were often hindered by expertise and resource constraints and absence of management support when using social media to promote vaccination, which was the cardinal public health directive during the COVID‐19 pandemic. Public managers' social media indifference can result in sparing or inefficient use of social media by the departments they lead [60]. Thus, PHSOs should implement programmes to foster a positive attitude towards the use of social media. Given that skill gaps in social media communication can contribute to inefficient use during public health crises [59, 61], PHSOs should prioritise the continuous training for their social media team to keep pace with the rapidly evolving social media environment. With enhanced capacity and skillset, PHSOs' social media communications managers and their teams will be better equipped to employ social media for public health messaging during future public health crises, confidently navigating the associated risks.

### 4.1 Implications, Strengths, Limitations and Future Directions

The results of this study have some important implications for the field of public health communication. Firstly, our findings underscore the significant impact of strategic public communication planning on pandemic management, particularly as it relates to the use of social media. PHSOs with a crisis communication plan are better prepared with the required resources and are more coordinated to respond to health emergencies, engendering transparency and public trust [62]. Secondly, the findings demonstrate the relevance of public engagement to PHSOs when using social media to communicate public health directives. People flocking to social media during pandemics is not new [10]. Therefore, PHSOs should have a solid plan for utilising social media to disseminate information. Finally, clearly defining measurable expectations for public health communication is critical given the potential benefits of effective execution of public health campaigns.

Our findings also have important implications for health equity. As social media has become a vital channel for disseminating public health information, inadequacies in the design and targeting of content can disproportionately disadvantage communities already experiencing social and structural inequities. Groups facing barriers such as limited country‐of‐residence language proficiency, low digital literacy or reduced trust in government institutions may be less likely to access, interpret or act on information shared through PHSO social media channels. These communication gaps risk reinforcing existing disparities in health outcomes, particularly during crises such as COVID‐19 when timely and trustworthy information is essential [63, 64]. Effective public health communication plays a central role in anchoring key health promotion principles related to community action and engagement [11]. Strengthening equity‐oriented social media practices—including culturally responsive messaging, multilingual content and co‐design with affected communities—is therefore critical to ensuring that public health communication supports, rather than undermines, equitable health outcomes.

One of the key strengths of our research lies in the rigorous approach taken to review the social media public communication component of the included pandemic response documents, achieved by using a standardised methodology and a quality appraisal framework guided by the Barcelona Principles 3.0 [27]. Furthermore, we assessed the implementation of this communication component through textual analysis to cross‐validate the results of the review process. According to Noble and Heale [65], data triangulation in qualitative research helps to overcome researcher biases. This jurisdiction‐level analytical review can be used as a model to evaluate the pandemic public communication plans of other countries practising a federal system of government similar to Australia's, with a view to enhancing communication coordination for future pandemics.

This study has limitations that present valuable opportunities for future research. Our study was limited to documents that were publicly available and that focused on public communication of public health directives in social media posts. We acknowledge that messages such as news updates and correction of false information are also important topics in which the public could be interested during pandemics [10]. We also focused our analysis on a broad range of public health directives. However, each directive had unique narratives during the COVID‐19 pandemic, particularly in relation to specific issues such as the timing of outbreaks involving different COVID‐19 variants. Also, there are opportunities to delve into subpopulation analysis, such as examining how the social media communication plans cater to different age groups or sociocultural affiliations. To attain a more comprehensive understanding of COVID‐19‐related public communication by Australia's PHSOs via social media, it is beneficial to conduct an in‐depth content analysis of their social media posts during the pandemic, supplemented by sentiment analysis of public responses and interviews with key PHSO communication staff. This would help triangulate this study's current findings and offer a more holistic view of the PHSOs' social media public communication initiatives. Finally, most of the jurisdictions have recently revised their pandemic management plans, incorporating lessons learnt during the COVID‐19 pandemic. Conducting similar analyses on an ongoing basis would be pertinent for continually strengthening public communication strategies for future pandemics.

## 5 Conclusion

This research aimed to assess the planning and implementation of social media public communication of COVID‐19 related public health measures in Australia. Our findings led us to conclude that the documents reviewed contained insufficient details to inform PHSOs to effectively employ social media for public engagement. It is anticipated that the findings will provide public health policymakers, practitioners and researchers with insights into the application of collaborative initiatives with the public through social media to propagate public health directives during pandemics.

## Funding

The authors have nothing to report.

## Ethics Statement

The authors have nothing to report.

## Consent

Informed consent was not required for this study because it did not involve human participants or animals.

## Conflicts of Interest

The authors declare no conflicts of interest.

## Supporting information


**Data S1:** hpja70179‐sup‐0001‐Supinfo.docx.

## Data Availability

The list of sources and their URLs of the data that support the findings of this study are available in Data S2 of the revised Supporting Information for review.
