# Supplementary material for: Planning and Implementing Social Media Communication in a Public Health Crisis: An Analytical Perspective
Source: Health Promot J Austr. 2026 Apr 5;37(2):e70179. doi: 10.1002/hpja.70179 (PMC13051203; doi:10.1002/hpja.70179)
Supplement: Supplementary file 1 — Data S1: hpja70179‐sup‐0001‐Supinfo.docx. [file HPJA-37-0-s001.docx]

Table of Content

[Supplementary Materials 2](#_Toc225167142)

[**Supplementary Material 1:** PHSOs’ Facebook communication of COVID-19 related posts 2](#_Toc225167143)

[**Supplementary Material 2:** COVID-19 pandemic emergency planning and management across jurisdictions in Australia 3](#_Toc225167144)

[**Supplementary Material 3A:** Data extraction template for COVID-19 pandemic management plans and post-COVID reports 4](#_Toc225167145)

[**Supplementary Material 3B:** Quality appraisal of the social media public communication plans 7](#_Toc225167146)

[**Supplementary Material 4:** Word frequency analysis of public health directives 9](#_Toc225167147)

[**Supplementary Material 5:** PHSOs’ COVID-19 pandemic management plans and first COVID-19 related activity on Facebook 11](#_Toc225167148)

# **Supplementary Materials**

### **Supplementary Material 1:** PHSOs’ Facebook communication of COVID-19 related posts

^Blue line indicates the PHSOs’ Facebook communication during the first 30 days of publishing their pandemic plans; Orange line indicates the PHSOs’ Facebook communication during the last 30 days of implementing their pandemic plans.^

### **Supplementary Material 2:** COVID-19 pandemic emergency planning and management across jurisdictions in Australia

**Table:** Relevant COVID-19 pandemic emergency management dates

| **Commonwealth and jurisdictions** | **Publication/activation of pandemic management plans** | | **End of pandemic emergency status declared** | |
| --- | --- | --- | --- | --- |
|  | **Date** | **Source** | **Date** | **Source** |
| Commonwealth AGDH | 18-Mar-20 | <https://www.health.gov.au/resources/publications/australian-health-sector-emergency-response-plan-for-novel-coronavirus-covid-19> | 20-Oct-2023* | <https://www.health.gov.au/news/end-of-covid-19-emergency-response> |
| ACT Health | 16-Mar-20 | <https://www.act.gov.au/__data/assets/pdf_file/0008/2356766/ACT-Chief-Health-Officers-Report-on-the-COVID-19-Public-Health-Emergency.pdf> | 29-Sep-22 | <https://www.covid19.act.gov.au/news-articles/covid-19-changes-from-30-september-2022> |
| NSW Health | 15-Mar-20 | <https://www.nsw.gov.au/sites/default/files/2021-04/emergency-management-subplan-human-influenza-pandemic.pdf> | 30-Nov-22 | <https://www.health.nsw.gov.au/Infectious/covid-19/Pages/public-health-orders.aspx> |
| NT Health | 19-Mar-20 | <https://health.nt.gov.au/__data/assets/pdf_file/0020/1179110/chief-health-officers-report.pdf> | 16-Jun-22 | <https://nds.org.au/news/nt-covid-19-update-northern-territory-s-public-health-emergency-declaration-will-cease> |
| QLD Health | 29-Jan-20 | <https://www.disaster.qld.gov.au/__data/assets/pdf_file/0020/340085/QLD-WoG-Pandemic-Plan.pdf> | 31-Oct-22 | <https://statements.qld.gov.au/statements/96421> |
| SA Health | 22-Mar-20 | <https://www.sahealth.sa.gov.au/wps/wcm/connect/a7539fe7-7d39-43e0-920d-94ac63983796/SAH_Viral_Respiratory_Disease_Pandemic_Response_Plan_final.pdf?MOD=AJPERES&amp;CACHEID=ROOTWORKSPACE-a7539fe7-7d39-43e0-920d-94ac63983796-nKP.9b1> | 24-May-22 | <https://www.premier.sa.gov.au/media-releases/news-items/covid-major-emergency-declaration-ends> |
| TAS Health | 17-Mar-20 | <https://www.audit.tas.gov.au/wp-content/uploads/2021-No-10-Report.pdf> | 30-Jun-22 | <https://www.dpac.tas.gov.au/divisions/ssmo/coronavirus/hoss_addresses/coronavirus/covid-19-update> |
| VIC Health | 16-Mar-20 | <https://www.aph.gov.au/About_Parliament/Parliamentary_departments/Parliamentary_Library/pubs/rp/rp2021/Chronologies/COVID-19StateTerritoryGovernmentAnnouncements#_Toc52275803> | 12-Oct-22 | <https://www.premier.vic.gov.au/changes-pandemic-management> |
| WA Health | 17-Mar-20 | <https://www.aph.gov.au/About_Parliament/Parliamentary_departments/Parliamentary_Library/pubs/rp/rp2021/Chronologies/COVID-19StateTerritoryGovernmentAnnouncements#_Toc52275804> | 4-Nov-22 | <https://www.wa.gov.au/government/covid-19-coronavirus> |

^*This study used 8 May 2023 date, the day WHO declared an ended the emergency status of pandemic. AGDH = Australian Government Department of Health and Aged Care; ACT = Australian Capital Territory; NSW = New South Wales; NT = Northern Territory; QLD = Queensland; SA = South Australia; TAS = Tasmania; VIC = Victoria; WA = Western Australia^

### **Supplementary Material 3A:** Data extraction template for COVID-19 pandemic management plans and post-COVID reports

**Section 1: Document characteristics**

1.1 Policy/document title

1.2 Policy/document type, including source and number of pages

1.3 Origin (national or jurisdictional)

1.4 Publication year (or date, if available)

1.5 Publisher organisation, including URL if available (i.e. please try and find the document online)

- Publisher's name:
- URL of document (if available):

1.6 Does the document describe a national or jurisdictional approach to COVID-19 pandemic management?

- Yes, continue to 2.1 Yes
- No, exclude the document and cease review of this document.

**Section 2: Data extraction**

2.1 Does the document describe alignment with PPRR emergency management framework (Holley & McArthur, 2022) for a pandemic and outline activities for each stage?

Yes No

- If yes, provide this here:
  - How many stages?

Please provide details of stages included in the document:

2.2 Does the document include and describe a public communication plan as part of the national or jurisdictional approach to COVID-19 pandemic management?

Yes (directly) Yes (indirectly) No

If yes, continue to 2.3,

2.3 Does the public communication plan explicitly state its purpose, aim, or vision?

Yes (directly) Yes (indirectly) No

If yes, provide this here:

2.4 Does the document include and describe the measures relating to the public communication for each stage mentioned in 2.1?

Yes Partially No

2.5 Does the public communication plan clearly identify its audience?

Yes No

If yes, provide this here:

2.6 Does the communication plan include specific (at-risk) target audience to address?

Yes No

If yes, provide this here: (all the groups of audience)

2.7 Does the communication plan provide for a section of the audience with considerable level of English language barrier?

Yes No

If yes, provide the context here:

2.8 Does the public communication plan explicitly include social media in its communication mix?

Yes No

If no, continue to 2.13,

If yes, provide this here:

2.9 Does the communication plan explicitly state the types of messages that social media channels would communicate?

Yes (directly) Yes (indirectly) No

If yes, state the types of messages that are referred to:

2.10 Does the communication plan explicitly state the frequency of messaging via social media channels?

Yes (directly) Yes (indirectly) No

If yes, provide this here:

2.11 Does the communication plan explicitly state its intention to amplify the reach of messaging by using relevant social media tools and functions?

Yes (directly) Yes (indirectly) No

If yes, state the context:

2.12 Does the communication plan explicitly state the social media channels to be used?

Yes (directly) Yes (indirectly) No

If yes, state the context:

2.13 Does the communication plan explicitly state engagement in two-way communication via social media channels?

Yes (directly) Yes (indirectly) No

If yes, state the context in which the two-way communication would hold:

2.14 Does the communication plan explicitly acknowledge risks and challenges associated with social media communication especially during a pandemic?

Yes (directly) Yes (indirectly) No

If yes, state the context:

References

Briggs, A. M., Persaud, J. G., Deverell, M. L., Bunzli, S., Tampin, B., Sumi, Y., …, & Slater, H. (2019). Integrated prevention and management of non-communicable diseases, including musculoskeletal health: a systematic policy analysis among OECD countries. *BMJ Global Health*, 4(5), Article e001806.

Holley, A., & McArthur, T. (2022). PPRR and AIIMS: A whole-of-government strategy in NSW. *The Australian Journal of Emergency Management*, 37(3), 65-74.

Mahimbo, A., Seale, H., & Heywood, A. E. (2017). Immunisation for refugees in Australia: a policy review and analysis across all States and Territories. *Australian and New Zealand Journal of Public Health*, 41(6), 635-640.

### **Supplementary Material 3B:** Quality appraisal of the social media public communication plans

The tool below is based on criteria developed in the literature (Briggs et al., 2019; Stone et al., 2022). Definitions for assessment criteria are:

- *Fulfilled*: all the mentioned criteria are addressed,
- *Room for improvement*: some but not all the criteria are addressed,
- *Not fulfilled or weak*: no criteria are addressed,

** if the response to the criteria is not stated, assume "not fulfilled or weak".

**Table:** Quality appraisal framework

| Domain descriptions (domain 'A' not included) | Assessment criteria | | |
| --- | --- | --- | --- |
|  | Fulfilled or strong (2) | Room for improvement (1) | Not fulfilled or weak (0) |
| A: Background and 'case for social media' | | | |
| The introduction section, justifying social media communication, references: |  |  |  |
| • evidence from contemporary literature; and |  |  |  |
| • (citation of) compelling scientific grounds; and |  |  |  |
| • verifiable sources of data or authorities. |  |  |  |
| B: Public opportunity | | | |
| There is evidence that the plan: |  |  |  |
| • is informed by meaningful consultation (e.g. surveys, polls, etc.) with a broad range of stakeholders; and |  |  |  |
| • integrates evidence from consultation outcomes. |  |  |  |
| C: Goals | | | |
| The goals/objectives of the plan are: |  |  |  |
| • explicitly stated; and |  |  |  |
| • tangible enough to be evaluated; and |  |  |  |
| • oriented towards engaging the public and to persuade adoption of preventive measures. |  |  |  |
| D: Stakeholder orientation | | | |
| The plan identifies/recognises: |  |  |  |
| • target audience, including specific groups (e.g. First Nations, CALD, PLWD); and |  |  |  |
| • communication channels to reach each target audience; and |  |  |  |
| • communication needs of each target audience; and |  |  |  |
| • diversity in health literacy levels of the target audience; and |  |  |  |
| • specific metrics for each target audience; and |  |  |  |
| • potential conflicting sources of information that the target audience may access; and |  |  |  |
| • target audience as not passive hearers but active stakeholders, and |  |  |  |
| • opportunities to consult various target audience for the co-design of tailored messaging. |  |  |  |

**Table:** Quality appraisal framework (continued)

| Domain descriptions (domain 'A' not included) | Assessment criteria | | |
| --- | --- | --- | --- |
|  | Fulfilled or strong (2) | Room for improvement (1) | Not fulfilled or weak (0) |
| E: Time allocation | | | |
| The plan outlines: |  |  |  |
| • a probable calendar that schedules messaging and other campaigns; and |  |  |  |
| • time for different categories of messaging (e.g., situational information, important health messages, updates of case numbers and deaths); and |  |  |  |
| • time for listening and interacting with the public (e.g., enquiries, polls, competitions, etc.); and |  |  |  |
| • time for reporting the performance of social media activities (e.g. daily, weekly, etc.). |  |  |  |
| F: Measurement and evaluation | | | |
| The plan considers: |  |  |  |
| • the outputs based on quantitative metrics (e.g., reach, likes, shares, views, etc.); and |  |  |  |
| • the outcomes based on qualitative metrics (e.g., comments, sentiments, etc.); and |  |  |  |
| • the potential impact based on public awareness, attitude, or opinion data; and |  |  |  |
| • the frequency of measuring outputs, outcomes, and impact (e.g. daily, weekly, monthly, etc.); and |  |  |  |
| • the reporting of measured outputs, outcomes, and impact (e.g. daily, weekly, monthly, etc.). |  |  |  |
| G: Obligations | | | |
| The plan explicitly specifies and assigns: |  |  |  |
| • roles and responsibilities of implementers of activities (e.g. messaging, measuring and evaluation, listening and interacting, etc.) |  |  |  |
| H: Resource considerations | | | |
| The policy considers: |  |  |  |
| • the provision for funding of social media messaging; and |  |  |  |
| • sourcing of spokespersons for social media messaging; and |  |  |  |
| • sourcing of social media influencers for social media messaging. |  |  |  |
| J: Integrity and transparency | | | |
| The plan reveals: |  |  |  |
| • the estimated resources supporting social media messaging (e.g. campaigns produced by hired external parties); and |  |  |  |
| • mitigation efforts to manage the potential risks associated with communicating about an evolving public health challenge; and |  |  |  |
| • human resource competences and organisational capacity to implement the plan; and |  |  |  |
| • procedures and guidelines that inform how the public should use the organisation’s social media platforms. |  |  |  |

References

Briggs, A. M., Persaud, J. G., Deverell, M. L., Bunzli, S., Tampin, B., Sumi, Y., …, & Slater, H. (2019). Integrated prevention and management of non-communicable diseases, including musculoskeletal health: a systematic policy analysis among OECD countries. *BMJ Global Health*, 4(5), Article e001806.

Stone, J. A., Flanders, K. J., & Can, S. H. (2022). Strategic communication? Measurement and evaluation of Twitter use among municipal governments. *Government Information Quarterly*, 39(4), Article 101755.

### **Supplementary Material 4:** Word frequency analysis of public health directives

**Supplementary Material 4a:** Weighted coverage of 12 public health directives in the analysed documents

|  | **Digital**  **tracing app** | **Contact**  **tracing** | **Cough** | **Distancing/**  **distance** | **Hand/**  **hands** | **Isolation/**  **isolate** | **Mask** | **Quaran**  **-tine** | **Test** | **Stay home/**  **work from home** | **Surface**  **hygiene** | **Vaccination/**  **vaccine** |
| --- | --- | --- | --- | --- | --- | --- | --- | --- | --- | --- | --- | --- |
| **COVID-19 pandemic management plans (weighted document coverage*)** | | | | | | | | | | | | |
| Common-  wealth | - | 0.02 | 0.01 | 0.01 | 0.01 | 0.05 | 0.01 | 0.04 | 0.01 | - | - | 0.06 |
| NSW | - | - | - | 0.10 | - | 0.09 | - | 0.13 | 0.01 | 0.04 | - | 0.07 |
| QLD | - | - | - | 0.01 | 0.01 | 0.03 | - | 0.01 | 0.01 | - | - | 0.03 |
| SA | - | 0.03 | 0.01 | 0.03 | 0.01 | 0.04 | 0.01 | 0.04 | 0.01 | - | - | 0.15 |
| TAS | 0.01 | 0.06 | 0.01 | 0.02 | 0.01 | 0.04 | - | 0.08 | 0.01 | 0.01 | - | 0.01 |
| VIC | - | 0.02 | 0.01 | 0.04 | 0.02 | 0.07 | 0.01 | 0.06 | 0.01 | - | 0.01 | 0.02 |
| WA | - | 0.01 | - | 0.06 | 0.01 | 0.05 | - | 0.09 | 0.01 | 0.03 | - | 0.06 |
| **Post-COVID reports (weighted document coverage*)** | | | | | | | | | | | | |
| Common-  wealth | - | 0.04 | - | 0.03 | - | - | 0.01 | - | - | - | - | 0.24 |
| ACT | 0.01 | 0.06 | - | 0.05 | 0.01 | 0.09 | 0.02 | 0.32 | 0.04 | 0.02 | - | 0.41 |
| NSW | - | 0.07 | - | 0.01 | 0.01 | 0.05 | 0.01 | 0.05 | 0.01 | 0.01 | - | 0.13 |
| NT | 0.01 | 0.02 | - | 0.01 | 0.01 | 0.01 | 0.01 | - | 0.01 | 0.01 | - | 0.02 |
| QLD | - | 0.01 | - | 0.01 | 0.01 | 0.02 | 0.01 | 0.02 | 0.02 | 0.01 | - | 0.23 |
| SA | 0.01 | 0.04 | - | 0.01 | - | 0.03 | 0.01 | 0.08 | 0.01 | 0.01 | - | 0.10 |
| TAS | - | 0.08 | - | 0.01 | 0.01 | - | - | 0.02 | 0.01 | - | - | 0.01 |
| VIC | 0.01 | 0.01 | - | 0.01 | 0.01 | 0.05 | 0.01 | 0.04 | 0.01 | 0.01 | - | 0.32 |
| WA | 0.01 | 0.03 | - | 0.02 | 0.01 | 0.04 | 0.01 | 0.18 | 0.01 | 0.01 | - | 0.30 |

^*Weighted document coverage represents the number of mentions of a public health directive in each analysed document as a percentage of all the words used in the document.^


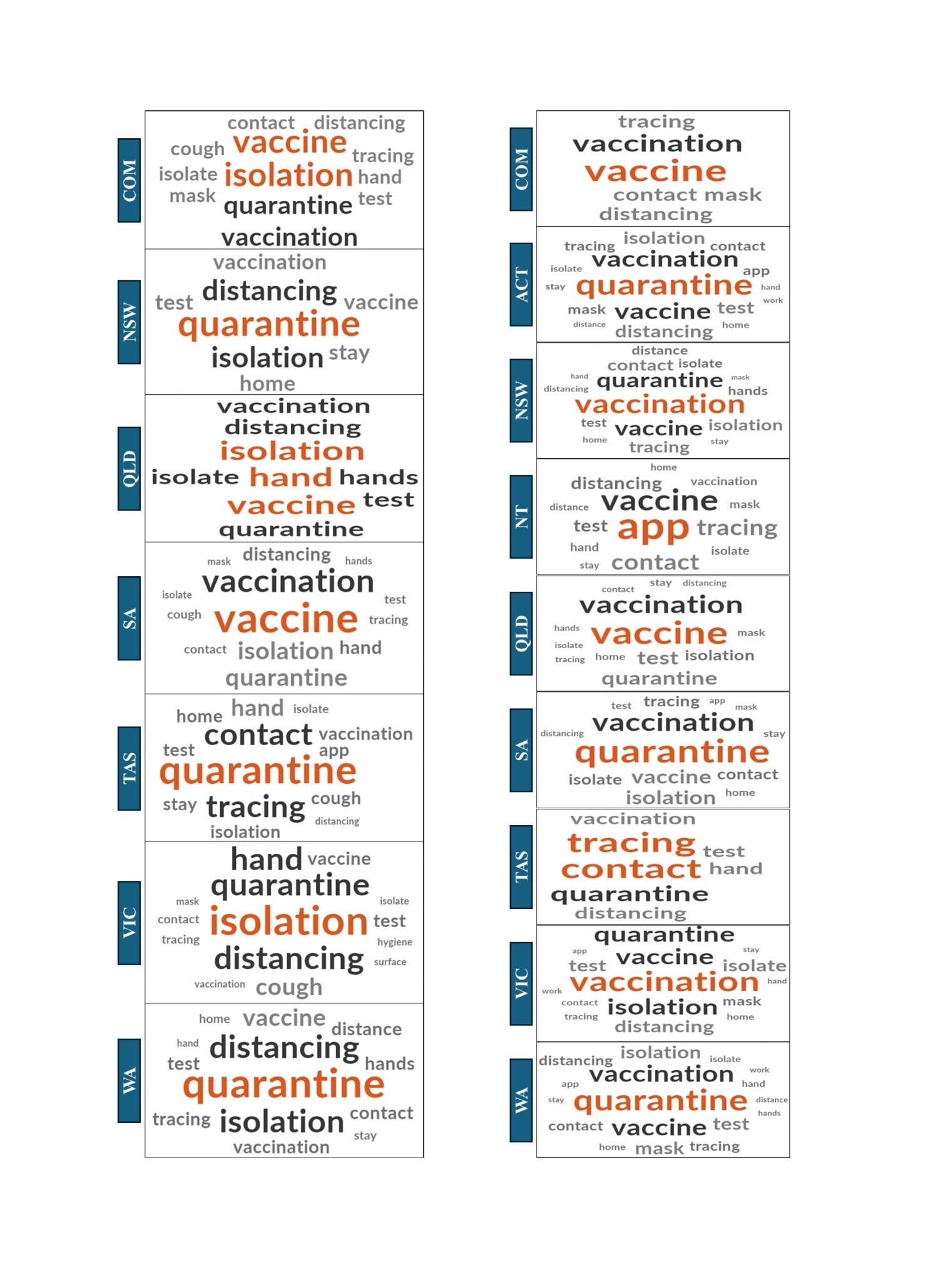


**Supplementary Material 4b:** NVivo Word Cloud of public health directives discussed in the pandemic management plans (left) and post-COVID reports (right) of individual jurisdictions

### **Supplementary Material 5:** PHSOs’ COVID-19 pandemic management plans and first COVID-19 related activity on Facebook

| **Australia’s Public Health Service Organisations** | **Title of Pandemic Management Plan** | **Dates** | | | | | |
| --- | --- | --- | --- | --- | --- | --- | --- |
|  |  | **Publication of plan** | **Emergency declaration** | **First COVID-19 case** | **Facebook account registration** | **First COVID-19 related Facebook post** | **End of emergency**** |
| Common-  wealth AGDH | Australian Health Sector Emergency Response Plan for Novel Coronavirus (COVID-19) | 18-Feb-2020 | 18-Mar-2020 | 25-Jan-2020 | 10-Jan-2013 | 21-Jan-2020 | 20-Oct-2023 |
| ACT Health | ACT Health Emergency and Epidemic Infectious Disease Plans | 29-Jan-2020* | 16-Mar-2020 | 12-Mar-2020 | 8-Dec-2011 | 24-Jan-2020 | 29-Sep-2022 |
| NSW Health | NSW Human Influenza Pandemic Plan: A Sub Plan of the NSW State Emergency Management Plan | 7-Jun-2018 | 15-Mar-2020^ | 25-Jan-2020 | 16-Apr-2014 | 22-Jan-2020 | 30-Nov-2022 |
| NT Health | COVID-19 NT Health Pandemic Plan | Early 2020* | 19-Mar-2020 | 4-Mar-2020 | 28-Apr-2010 | 24-Jan-2020 | 16-Jun-2022 |
| QLD Health | Queensland Whole-of-Government Pandemic Plan | Mar-2020 | 29-Jan-2020 | 29-Jan-2020 | 27-Mar-2012 | 27-Jan-2020 | 31-Oct-2022 |
| SA Health | SA Health Viral Respiratory Disease Pandemic Response Plan (including influenza, COVID-19, SARS & MERS) | Mar-2020 | 22-Mar-2020 | 1-Feb-2020 | 9-Sep-2011 | 24-Jan-2020 | 24-May-2022 |
| TAS Health | COVID-19 specific State Special Emergency Management Plan | 17-Mar-2020* | 17-Mar-2020 | 2-Mar-2020 | 15-Oct-2013 | 25-Jan-2020 | 30-Jun-2022 |
| VIC Health | COVID-19 Pandemic plan for the Victorian Health Sector | 10-Mar-2020 | 16-Mar-2020 | 25-Jan-2020 | 11-Nov-2011 | 25-Jan-2020 | 12-Oct-2022 |
| WA Health | Western Australian Government Pandemic Plan | 11-Mar-2020 | 17-Mar-2020 | 21-Feb-2020 | 12-Apr-2013 | 22-Jan-2020 | 4-Nov-2022 |

^AGDH = Australian Government Department of Health and Aged Care; *Activation dates; **See Appendix 1; ^First public health order (no classic emergency declaration made)^
